# Telehealth program for symptomatic COVID-19 patients in Mindanao, Philippines: a whole-of-system, pragmatic interventional study on patient monitoring from isolation facilities to community reintegration

**DOI:** 10.1186/s12939-024-02115-5

**Published:** 2024-02-03

**Authors:** Jaime Kristoffer Punzalan, Monserrat Guingona, Elgie Gregorio, Jhufel Ferraren, Mark Anthony Sta. Elena, Marvin Valaquio, Floro Dave Arnuco, Mary Germeyn Punzalan, Rosemarie Arciaga, Torres Woolley, Afdal Kunting, Dulce Amor Miravite, Fortunato Cristobal

**Affiliations:** 1 Ateneo de Zamboanga University, Zamboanga City, Philippines; 2 Zamboanga City Medical Center, Zamboanga City, Philippines; 3 Zamboanga City Health Office, Zamboanga City, Philippines; 4 James Cook University, Queensland, Australia

**Keywords:** Whole-of-society approach, Telehealth program, COVID-19, Preventive practices, Mental health

## Abstract

The COVID-19 pandemic is impacting individuals and society's physical and mental health. Despite the lack of any definite and effective therapeutic regimen, public health measures such as quarantine and isolation have been instituted to contain this pandemic. However, these mitigating measures have also raised issues regarding isolated patients' mental and psychological well-being. Several stakeholders were engaged in this approach, including the university, the local health office, the tertiary hospital, and the local communities. This intervention addresses concerns regarding the health status of isolated individuals due to COVID-19 infection, making the program available to anyone who agrees to participate. This was done through telehealth services delivered via phone calls and SMS. The university provided technical support and telehealth manpower through medical students. The local health unit manages the isolation facilities, while the referral hospital offers specialty care for isolated patients through teleconsultation. Finally, the local community is the one that reintegrates discharged patients into their communities. Three hundred forty-four (344) participants were provided seven sessions on telehealth education and tracking of their COVID-19 prescribed practices and mental health. The mean age of the patients was 37 years; half were females, and 15% had comorbidities. Regarding their mental health status, the level of depression dropped from 6% to 1% (*p*<0.0001), the level of anxiety dropped from 12% to 2% (*p*<0.0001), and the level of stress dropped from 3% to 0% (*p*<0.0001) from the first day of admission to 2 weeks after discharge. Moreover, a general trend of statistically significant increase in various practices was noted: wearing face masks, physical distancing, disinfecting frequently held objects, hand hygiene, and self-monitoring for COVID-19 symptoms. Those with progressing symptoms of COVID-19 were referred immediately to the referral hospital. There were also no reports of complications of co-morbidities during their stay in the isolation facilities or social isolation upon community reintegration. The study concludes that telehealth services have the potential to address many challenges in providing continuous healthcare services to isolated patients until they are reintegrated into their community. Furthermore, a whole-of-society approach is necessary to provide holistic care to patients affected by the pandemic.

## Background

The COVID-19 pandemic represents a major 21st-century unprecedented event, impacting individuals and society's physical and mental health [1]. The high transmissibility of the COVID-19 virus, with the resultant high mortality rate, has led the government and other institutions to institute mitigating measures to contain the rapid spread of this virus [2, 3]. This was done through community lockdowns, cancellation of social gatherings, instituting social distancing, and wearing a face mask and shield. Unfortunately, most individuals felt that the crisis had greatly restricted their daily life, including daily routines and essential activities [4]. The establishment of isolation facilities, combined with social distancing, has also created unexpected health consequences. By default, these measures have caused some mental health problems. Several studies have explored the psychological and mental health impact of the drastic changes brought about by the pandemic crisis. The result was consistent among the studies in different countries: approximately 20% to 30% of participants experienced mild to severe anxiety levels. Approximately 40-50% of the participants had increased levels of depression [5–8].

The paper by Ramírez-Ortiza on the mental health consequences of the COVID-19 pandemic associated with social isolation showed that there are multiple associated psychological disturbances, ranging from isolated symptoms to complex disorders with marked impairment of functionality, such as insomnia, anxiety, depression, and posttraumatic stress disorder [9]. Telehealth consultation and education can be provided to patients confined in restricted isolation facilities. This platform was explored through a systematic literature review by Langarizdeh et al. [10]. This preview reported that telemental health services have multiple capabilities and technologies for delivering interventions related to mental health services. It was also considered advantageous and inexpensive compared to in-person services. Concerning the COVID-19 crisis, Zhou et al. and Reay, Looi, and Keightley both affirmed the use of mental health services through telehealth platforms as feasible and appropriate for the delivery of mental health services to patients in isolation, including their family members and health providers, during the pandemic [11, 12].

All that said, telehealth can also provide flexibility while giving patients access to a healthcare provider 24/7. One can access healthcare if one has access to a phone, tablet, or computer [13]. Integrating telehealth with other medical services provides limited access and allocation to health information and resources. Furthermore, it promotes trust, physician-patient engagement, and patient satisfaction with the health service provided [14]. Telehealth is important for urban and rural areas and offers unlimited virtual visits, psychotherapy, consultations, and other medical or health services [15]. A review by Monaghesh et al. concluded that telehealth technology improves health services while protecting patients and providers from COVID-19 exposure [16]. This was especially beneficial to isolating patients, as it helped minimize their risk of COVID-19 transmission while they kept communication lines open and connectivity for emotional support [17, 18]. Continuous care to patients and the communication links facilitated through telehealth services are very important, especially during a crisis where geographical distance and the threat of contamination are actual. Telehealth services can be used to prevent, diagnose, treat, and control diseases during the COVID-19 outbreak [19].

These services are practical, feasible, and appropriate to provide conversation links between patients and their family members or for referrals to healthcare providers [11]. Knowledge and information delivered via telehealth services to confined, isolated patients can be therapeutic to avert the development of full-blown mental disorders [20]. There is compelling evidence to suggest that telehealth may significantly advance health care. However, the feasibility and application of telehealth in resource-limited settings and low- and middle-income countries are daunting. Existing gaps must be bridged to avail of its potential benefits to deliver health care to the world’s expanding population. Therefore, this paper aims to determine the impact of a whole-of-society approach in establishing a telehealth education and consultation program in terms of preventive quarantine practices and mental health status of COVID-19-positive individuals confined in isolated facilities in Zamboanga City, Mindanao, Philippines.

## Methodology

### Research design

This research project is a pragmatic one-group quasi-experimental study implemented in the isolation facilities of Zamboanga City. Monitoring and evaluation were performed by means of Information and Communications Technology (ICT). The local university's assigned postgraduate interns (PGIs) continuously provided telehealth services and continued monitoring the patient’s home quarantine practices and mental health status. Contact details of the potential participants were first collected by the Health Facility Teams (HFT) assigned to the isolation facilities. The HFTs are healthcare workers under the local health unit who manage the isolation facilities and monitor the isolated patients. The recruited participants were enrolled in the Telehealth Education and Consultation program. On discharge from these isolation facilities, these patients were still monitored while in home-based quarantine for one week before they were released to reintegrate into the community. The Health District Team (HDT) facilitated this reintegration into the community. The HDTs were community health workers who monitored patients as they were discharged from isolation facilities and transitioned to home quarantine.

In planning for the project, postgraduate interns were recruited to provide the manpower to run the telehealth services. Before the program's implementation, technical issues, such as communication expenses and signal problems, were proactively tackled. Mitigating measures were implemented to address these concerns, including communication allowances and signal mapping for isolation facilities. There was no face-to-face contact between the PGI and the patients. This project is also linked with the COVID-19 Center of the tertiary hospital to ensure a seamless communication link in case there is a need for further referral of the patient’s concern to service consultants. The tertiary hospital is the only COVID-19 referral center in the region. It was also the receiving facility for isolated patients with worsening symptoms.

### Respondents

The target participants for this program were individuals who were confirmed positive for COVID-19. They were confined in 3 of the isolation facilities of the city. Upon admission to the isolation facilities, contact details and other demographic data are first collected by the assigned HDT. Consent to participate in this project was also sought. The HDT subsequently forwarded these contact details and demographic data to the telehealth personnel of the local university. It was made clear to the patient that participation in the program was voluntary. They were assured that they would still receive standard medical care whether they denied or withdrew their participation. These patients were then designated to a PGI assigned at a specific isolation facility. The PGI contacted the assigned patient, reviewed their consent to participate, and took their general demographic information. Explanations about the project and its purpose were also provided. Once consented, the PGI initiated the telehealth education and consultation as scheduled until discharge. Aside from providing telehealth services, a mental health assessment is performed upon initial entry and discharge from the isolation facility.

### Research setting

The study was implemented in three isolation facilities identified by the City Health Office. These isolation facilities were chosen because of their bed capacity, ease of communication, and availability of continuous health services from the start to the end of the program.

Facility A is located near the City Hall and City Health Office. It has the highest bed capacity, with 110 available beds. It has 36 isolation rooms, each accommodating up to 3 individuals. A double wood paneling was used to separate each individual in every room. Some of the rooms also have air conditioning units. However, windows were utilized to promote room ventilation, maximizing the breeze from the nearby ocean. Moreover, each room also has its comfort room. Most of the general population of COVID-19-positive patients is isolated in this facility. It has a good network signal for communication and is accessible to most people. Additionally, it has a beautiful ambiance, with the waterfront facing the wharf where inter-island ferry boats dock and exit continuously daily.

Facility B has the lowest bed capacity, with only 50 beds. It is in the uptown area of the city. It is a renovated 2-story public office building to accommodate the needs of individuals for isolation. The individuals are separated with wood paneled dividers and can be accessed through commercial-used curtains. The interior has a moderately high ceiling and is well-ventilated with large windows. It has a common comfort room per floor for the patients accommodated in the said establishment. Most individuals isolated in this facility were uniformed personnel and government employees. There are a few locations within the facility where the network signal could be better, but it did not significantly hinder the communication lines.

Facility C has a 60-bed capacity and lies more than 10 kilometers from the Zamboanga City Hall. The accommodation is through separate villas rather than a building with rooms. Each villa can accommodate about 5-6 individuals for isolation. Dividers were also placed to separate each individual from each other. Additionally, each villa has its standard comfort room. The villas are also well-ventilated, with the sea breeze coming through the windows. Most of the individuals isolated in this facility were from the West Coast area of the city. It offers a good network signal throughout the area. Nevertheless, there were times when operations were temporarily put on hold due to renovations and disinfection activities within the center, but this center is famous for its green ambiance.

### Telehealth education

Telehealth education is delivered through voice calls using mobile phones. It primarily includes transferring information from the health provider, the PGI, to the participants, the COVID-19-positive patient. The health education content focuses mainly on coronavirus disease 19 preventive practices and mental health. The sessions are divided into 2 phases: the isolation facility phase and the community phase. Before implementing this program, the PGI were given training on telehealth system operation, mental health assessment, and virtual simulation rehearsals focusing on cellphone etiquette. A script guides how to start and end communication with the participants for each session. Table 1 below shows the module content for telehealth education.

**Table 1:** Telehealth education module plan

	**Isolation Facility**					**Home Quarantine**	**Community Reintegration**
**Topic**	Session 1 (Day 3)	Session 2 (Day 4)	Session 3 (Day 5)	Session 4 (Day 6)	Session 5 (Day 7)	Session 6 (Day 11)	Session 7 (Day 21)
**Introduction**	Initial Opening – introduction Ask General Health Status including COVID-19 symptoms	Ask General Health Status including COVID-19 symptoms	Ask General Health Status including COVID-19 symptoms	Ask General Health Status including COVID-19 symptoms	Ask General Health Status including COVID-19 symptoms	Ask General Health Status including COVID-19 symptoms	Ask General Health Status including COVID-19 symptoms
**Health Education on Preventive Practices**	General Hand Hygiene Practices	Protective Personal Equipment	Regular and frequent health monitoring	COVID-19 vaccine information and acceptability	Summary of good practices	Home quarantine practices	Possibility of COVID-19 reinfection
	Physical Distancing	Cough etiquette	Disinfection of frequently held household items	Identification of high-risk individuals at home		Close contact monitoring	Work-related COVID-19 preventive practices
**Mental Health Education**	Introduction to mental health	Common signs and symptoms of mental health disorders	Causes of mental health disorders	Encouraging to help-seeking			
**Closing**	Schedule next session	Elicit questions and clarifications	Elicit questions and clarifications	Elicit questions and clarifications	Elicit questions and clarifications	Elicit questions and clarifications	Elicit questions and clarifications
	Elicit questions and clarifications				Orientation for Monitoring and Evaluation		

### Telehealth Consultation

Telehealth consultation is a medical consultation with appropriate assistance extended to participants through voice calls. The communication flow was between the participant and the PGI. They may need to refer other patient concerns to the HFT and the assigned specialist from the tertiary hospital. Disposition largely depended on the gravity of these concerns. If the participant's concern is a COVID-19-related presenting symptom, the PGI referred the participant to the HFT for further assessment. If the concern was a health complaint other than COVID-19 presenting symptoms, the PGI referred the participant to the Internal Medicine Department of the tertiary hospital. If the participant's concern was mental health-related, the PGI referred the participant via telehealth consultation to the Psychiatry Department of the tertiary hospital. This referral system network ensures the isolated patient can access all available health services. If further assessment, management, and treatment were needed, the participant was referred to the HFT stationed in each isolation facility for further disposition.

### Research instruments

The COVID-19 prescribed quarantine practices that were gathered for this study were wearing face masks, observing physical distancing, disinfecting handheld items, frequent handwashing, and regular self-monitoring. This information was limited to self-reported practices due to COVID-19 protocol restrictions for on-site observations. A Likert scale with always, sometimes, and never responses was obtained weekly through recall.

In our study, we conducted a mental health assessment using the Depression, Anxiety, and Stress Scale-21 or DASS-21. This scale is a concise version of the DASS-42, a self-reported instrument designed to evaluate negative emotional states, specifically depression, anxiety, and stress. Research has demonstrated high internal consistency of the three scales within the DASS, exhibiting the ability to provide meaningful distinctions. The DASS-21 utilizes a 21-item questionnaire with seven questions about the three mental health states. Respondents were given the options of 'Never,' 'Sometimes,' 'Often,' and 'Always' for each question, with corresponding points assigned to these responses. The cumulative score for each scale helps determine the severity level, as outlined in Table 2. Notably, the use of DASS-21 did not require permission to employ this questionnaire, as it is freely available in the public domain [21]. The translation process of the DASS-21 into Filipino was conducted locally during our study.

**Table 2:** Depression, Anxiety, and Stress Scale (DASS)-21 scores according to the severity

**Subscale**	**Depression**	**Anxiety**	**Stress**
**Normal**	0-4	0-3	0-7
**Mild**	5-6	4-5	8-9
**Moderate**	7-10	6-7	10-12
**Severe**	11-13	8-9	13-16
**Extremely Severe**	14+	10+	17+

### Data gathering procedures

The HFTs first collected demographic data and other contact details. The collected data included the patient’s name, age, sex, cellphone number, address, co-morbidities, and COVID-19 presenting symptoms. This was done upon admission of the patient into the isolation facilities. This information was forwarded to the local university faculty, who randomly assigned the participant to a PGI. The latter initiated contact with the assigned participant. The PGI first verified the information and asked for additional information, such as the reason for their isolation and risk assessment for hypertension, diabetes mellitus, and COVID-19. The focus on hypertension and diabetes mellitus was done because the university already has a prepared module for such co-morbidities. In addition, consultations for other co-morbidities were done with resident physicians from the Internal Medicine Department of the tertiary hospital when necessary. This information served as baseline data needed in structuring the telehealth education module. COVID-19 preventive practices and a mental health assessment were also gathered. In cases where a participant registered a 'Moderate' to 'Extremely Severe' severity level in any of the Depression, Anxiety, or Stress subscales, they were asked for their consent for a referral to a psychiatrist at the tertiary hospital. If the participant declined this referral, the assigned PGI promptly notified the HFTs of the participant's status, with due consideration for the participant’s consent. The COVID-19 presenting symptoms, preventive practices, and mental health assessment were assessed every seven days until discharge from the isolated facilities. These participants were still tracked and monitored until they had completed the mandatory community home quarantine before full release to circulate and report back to work. The schedule of preventive practices, mental health, and quality of life assessments can be found in Table 3. When the participant cannot be contacted, the PGI referred the patient to the HDTs to follow up with the patient concerning their participation in the program.

**Table 3:** Schedule of preventive practices, mental health, and quality of life assessments

**Phase**	**Schedule**	**Activity**	**Content**
**Isolation Facility**	Day 1	Profiling	Consent Demographic Data COVID-19 Symptoms
	Day 2	Assessment	COVID-19 Preventive Practice Mental Health Assessment
	Day 3	Telehealth Education Session	Session 1
	Day 4		Session 2
	Day 5		Session 3
	Day 6		Session 4
	Day 7		Session 5
	Day 8	Monitoring	COVID-19 Preventive Practice Mental Health Assessment
**Home Quarantine**	Day 11	Telehealth Education Session	Session 6
	Day 15	Monitoring	COVID-19 Preventive Practice Mental Health Assessment
**Community Reintegration**	Day 21	Telehealth Education	Session 7
	Day 28	Monitoring	COVID-19 Preventive Practice Mental Health Assessment

The attendance of participants in telehealth education was recorded. Additionally, the number and content of telehealth consultations were recorded.

### Data analysis

Frequency and percentages were used to summarize the demographic data, COVID-19 prescribed practices, and mental health assessment. The number of participants who self-reported with the correct practice for each prescribed practice was noted. For the mental health assessment, binomial data were used to categorize the participants as either normal (normal to mild levels) or high (moderate to extremely severe) levels of depression, anxiety, and stress. A McNemar test was utilized to determine if there were any significant changes in the number of participants performing correct quarantine practices and with normal levels of depression, anxiety, and stress in comparison to the Day 2 results. Bar graphs were also used to visualize the study variables' changes better.

## Results

This study was implemented from February 1 to July 28, 2021. During this study, the city experienced a peak rise in confirmed COVID-19 cases (from April to June), and the city was again put into a heightened lockdown. The number of participants enrolled in the program can be seen in Table 4. Several reasons for loss to follow-up were observed, including individuals desiring rest during their isolation period, lacking interest in the program, or being preoccupied with work commitments after completing the quarantine period.

**Table 4:** Total number of patients admitted to the isolation facility who consented to participate in the program and completed the 28-day program

	**Frequency (%)**
**Number of admitted patients into the facility**	1368
**Participants who consented to join the program**	460 (33.6%)
**Participants who completed the 28-day program**	344 (75%)
**Drop-out rate**	126 (25%)

### Demographic data

The demographic data of participants enrolled in the program and completed the 28-day program is shown in Table 5. The mean age of the participants was 37 years old, with 138 (40%) of the participants between the ages of 21-30. Only 19 (5%) participants were 18-20 years old. Sixty-six patients were aged 31-40 years old and 41-50 years old. Only 33 (9%) participants were within the range of 51-60 years old. Twenty-three (6%) participants were 61 years old and above. More than half of the participants, or 55%, were female.

**Table 5:** Demographic data of the isolated individuals from February to July 2021 (*n*=344)

	**Frequency (%)**
**Age**	
**<21-30**	147 (45%)
**31-40**	66 (19%)
**41-50**	66 (19%)
**51-60**	33 (9%)
**61-70**	15 (4%)
**71- >**	8 (2%)
**Mean Age**	37 years old
**Sex**	
**Female**	189 (55%)
**Male**	156 (45%)
**With Comorbidities**	
**Hypertension**	30 (9%)
**Diabetes mellitus**	13 (4%)
**COPD/asthma**	8 (2%)
**Multimorbidity**	22 (6%)
**Isolation Facility**	
**Facility A**	203 (60%)
**Facility B**	68 (20%)
**Facility C**	71 (20%)

Only 30 (9%) participants had hypertension, and 13 (4%) had diabetes mellitus. Eight participants, or 2%, were previously diagnosed with COPD or asthma, while thirteen participants (4%) had multiple co-morbidities. There were only a few participants with co-morbidities in this study. Perhaps this is because high-risk individuals are likely to have severe symptoms, so they were admitted to the COVID-19 referral facility centers of ZCMC instead of the isolation facility.

Among the isolation facilities, most participants, or 60%, were confined in Facility A, 20% in Facility B, and 20% in Facility C. This unequal admission distribution is because Facility A has the highest number of beds (110 beds) compared to Facilities B and C, which have only 60 and 50 beds, respectively. Additionally, Facility A was a preferred center beside the scenic waterfront and was previously an iconic hotel. In addition, Facility A accommodates new COVID-19-confirmed patients and step-down cases from the tertiary hospital.

### COVID-19 preventive practices

**Fig. 1:**
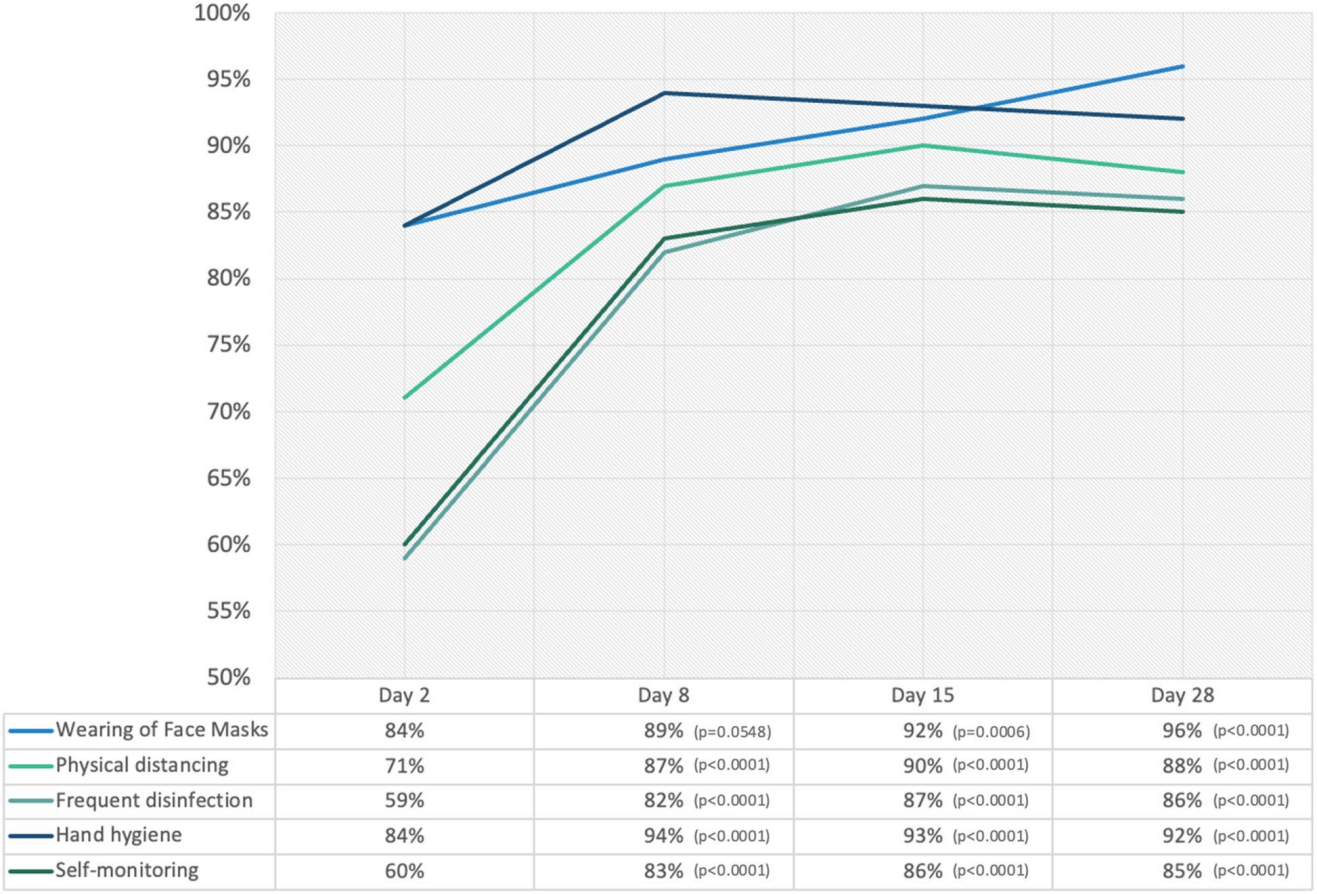
Bar graph of the percentage of the participants practicing specific COVID-19 preventive practices from Day 2 to Day 28 of the program (*n*=344)

The COVID-19 prescribed practices of the participants were assessed on Days 2, 8, 15, and 28, counting from their 1^st^ day of admission (Fig. 1). Day 2 assessment served as the baseline data as the participant's first contact with the program. Day 8 assessment was conducted after completing the 5-day sequential telehealth education sessions. On day 15, an assessment was performed when the participant started the home quarantine phase of the program. Upon discharge from the isolation facility, the participants still had to complete the home quarantine period of 7 days. The Day 28 assessment was performed when the participants were allowed to leave home to integrate back into their work outside the home entirely. All data gathered were self-reported information.

The study revealed the self-reported compliance of participants to COVID-19 preventive practices during their isolation and reintegration phases. Before their admission to the isolation facility, a proportion of participants were already correctly wearing face masks (84%) and practicing physical distancing (71%). Furthermore, frequent hand hygiene was common among participants before admission (84%). This initial compliance suggests a certain level of public awareness regarding the significance of these practices. However, the study noticed a consistent increase in adherence to these practices during isolation, with a statistically significant improvement from Day 15 onwards. This may point to the positive impact of telehealth education in reinforcing the importance of proper face mask usage and physical distancing as effective preventive measures against COVID-19.

Disinfection of frequently held items, such as cell phones and doorknobs, initially had a lower compliance rate (59%). However, there was a notable increase, with 82% of participants adopting this practice by Day 8. A significant improvement from Days 8, 15, and 28 compared to the baseline on Day 2 underscores the potential of telehealth in instilling the importance of disinfection. Similarly, self-health monitoring for COVID-19-related symptoms had a lower initial compliance rate (60%). Nevertheless, there was a substantial increase, with 83% of participants reporting self-monitoring on Day 8, which persisted until Day 28, with 85% of participants monitoring themselves for symptoms. 

### Mental health of isolated individuals

**Fig. 2:**
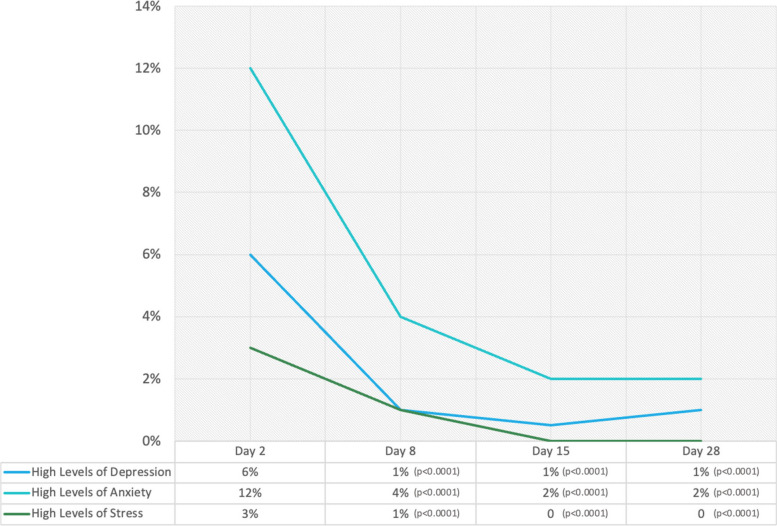
Percentage of the participants with high levels (moderate to extremely severe) of depression, anxiety, and stress from Day 2 to Day 28 of the program (*n*=344)

Along with monitoring COVID-19 preventive practices, participants were assessed for their mental health on days 2, 8, 15, and 28 (Fig. 2). Day 2 assessment was used as the baseline data for comparison of their levels of depression, anxiety, and stress. The tool used was DASS-21. On Day 8, the mental health assessment was performed while the participants were still in the isolation facility but preparing for discharge. The Day 15 assessment was performed when the participant was under home quarantine, while the Day 28 assessment was performed when they were free to return to their workplace.

At the outset of the isolation facility program, a small percentage of participants (6%) reported experiencing symptoms of depression, likely influenced by the apprehension of impending isolation and loneliness. However, by Day 8, the number of participants with depression symptoms significantly decreased to only two (2) and continued to decline to one (1) on Days 15 and 28. Notably, none of the participants reported homicidal or suicidal ideation, and these depressive symptoms were not reported during home quarantine and community reintegration.

Conversely, a higher proportion of participants reported experiencing anxiety at the beginning of their isolation, probably driven by the fear of their symptoms progressing into a severe COVID-19 condition. However, the reassurance provided through telehealth education sessions may have led to a notable decrease in anxiety levels, with only 4% experiencing anxiety on Day 8. This trend further diminished to 2% on days 15 and 28, and these reductions were statistically significant. Regarding stress, only a few participants (3%) reported stress upon entering the isolation facility, and this stress level decreased to 0% on Days 15 and 28. One hypothesis is that this change in stress levels may be attributed to a shift in participants' perspectives, from viewing isolation as punitive to considering it a period of rest and relief from work-related stressors. Notably, the absence of mental health symptoms persisted as participants transitioned back to their homes and communities.

## Discussion

Telehealth has emerged as a versatile and crucial tool in promoting various preventive practices during the COVID-19 pandemic. These practices encompass remote screening, care, treatment, monitoring, surveillance, detection, prevention, and mitigation of healthcare-related impacts indirectly associated with COVID-19 [22–24]. The literature has highlighted various telehealth consultation initiatives. These telehealth platforms have enabled remote screening for COVID-19, allowing healthcare providers to effectively assess patients' symptoms and exposure history from a safe distance [25, 26]. This not only aids in promptly identifying potential cases but also significantly reduces the risk of exposure within healthcare settings, effectively triaging patients for further care [16, 26]. In addition, telehealth has facilitated remote care, particularly for patients with mild to moderate COVID-19 symptoms. These individuals can be safely monitored from the comfort of their homes. Such an approach not only conserves valuable personal protective equipment (PPE) but also significantly reduces the exposure of healthcare workers to potentially infected patients [22].

A significant gap in the existing literature is the lack of emphasis on telehealth education [16, 22, 23, 27]. Effective prevention requires the practical application of preventive measures and informed decision-making by individuals regarding their health and safety. Telehealth platforms should serve as essential tools for educating individuals about the virus, preventive measures, and when to seek medical care. Moreover, telehealth services may have played a key role in reinforcing preventive measures. These include promoting practices such as wearing face masks, maintaining physical distancing, and emphasizing the importance of frequent handwashing and disinfection, all of which have been essential in breaking the chain of virus transmission. Future research and policy considerations should address this omission to ensure a comprehensive approach to pandemic prevention.

In the context of mental health, telehealth has provided a crucial avenue for individuals in isolation to seek emotional support, counseling, and guidance. The COVID-19 pandemic has not only inflicted physical health concerns but also significant mental health challenges. The rigors of isolation measures, disease progression, and symptoms can cause depression, anxiety, and stress in those who contract the virus [4].

Several studies in the Southeast Asian region have investigated the impact of COVID-19 on the mental health of patients. In particular, a study in Thailand during the pandemic's early stages revealed that hospitalized COVID-19 patients exhibited relatively low levels of anxiety and depression, with no discernible differences among demographic groups [28]. Likewise, the research conducted in Malaysia revealed a relatively low prevalence of anxiety among stable COVID-19 patients hospitalized. In contrast to global and regional trends, anxiety here was relatively low [29]. On the other hand, the study conducted in Vietnam, focusing on patients isolated in concentrated camps, were noted to have high rates of anxiety and depression [30]. Specific demographic groups, including older patients, women, primary income earners, and those grappling with physical symptoms, bore the burden of this emotional turmoil. Moreover, patients classified in the High Anxiety and Depression (HAD) group exhibited an increase in irritability. They reported diminished satisfaction with their living conditions compared to the Low Anxiety and Depression (LAD) group [30]. Several factors may influence the improvement of depression, anxiety, and stress levels after quarantine or isolation. These include daily stress, negative repetitive thinking, loneliness, detachment, and the presence of family. Numerous studies have identified these factors as having an impact on levels of depression, anxiety, and stress [31, 32]. On the other hand, this paper focused on providing telehealth programs to people dealing with isolation during the peak of the pandemic. At first, they experienced high levels of depression, anxiety, and stress. However, these levels gradually decreased as they moved from quarantine to reintegration with their community. Although one study in Indonesia found significantly lower rates of depression, anxiety, and stress post-quarantine compared to pre-quarantine [33], even without any specific intervention, the introduction of telehealth education and consultation may still have contributed to the improvement of the mental health status of the individuals.

The following studies collectively address the rapid implementation of telehealth programs in the context of the COVID-19 pandemic, focusing on its impact on mental health services. In Melbourne, Australia, clinicians treating individuals with severe personality disorders successfully transitioned to telehealth, reporting increased appointment reliability and acknowledging the benefits of telehealth despite concerns about privacy and interaction quality [34]. Clients with borderline personality disorder also adapted to telehealth, with most experiencing few technical issues and expressing interest in its continued use [35]. Similarly, a psychiatric practice in southeast Texas quickly adopted telepsychiatry during the pandemic, noting receptive patient engagement and the need for careful safety planning and professional boundaries [36]. The evidence from various studies indicates that telehealth is a practical and feasible mode of delivering mental healthcare, with phone and videoconferencing therapies demonstrating effectiveness and patient acceptance [12]. However, what truly distinguishes this study is the potential of telehealth in delivering mental health services intricately interwoven with the pandemic response and the focus on non-psychiatric individuals. A sustained commitment to telehealth education and consultation can reshape the delivery of psychological services to specific patient groups and at-risk individuals.

The findings presented in this study hold significant implications for policy and practice in the context of public health and healthcare delivery during a pandemic, particularly concerning telehealth. First, telehealth initiatives in this study highlight the necessity of integrating telehealth into pandemic response plans. Policymakers should prioritize the development of a whole-of-society approach in telehealth infrastructure that can cater to a diverse range of healthcare needs, including general medical consultations, mental health support, and monitoring of at-risk individuals. This integration should involve the establishment of clear guidelines for its implementation, the accreditation of healthcare providers, and the inclusion of telehealth services in the insurance coverage [37, 38]. Telehealth can be a valuable tool for conserving resources, reducing exposure risks, and ensuring that healthcare services reach those in need, even in the most challenging circumstances [39].

Additionally, the study underscores the contribution of telehealth education and consultation in addressing physical health concerns and psychological well-being. Public health policies should encourage developing and implementing telehealth programs, which can be crucial in patient education and reassurance during isolation or quarantine periods [19]. Initiatives to enhance the skills of healthcare professionals in delivering telehealth education should be actively supported [40]. In practice, the positive outcomes observed among isolated individuals should inspire healthcare providers to employ telehealth not only for medical care but also to address the heightened levels of depression, anxiety, and stress that can accompany pandemic-related isolation. This notion is further exemplified by a study conducted in Austria, which divulges a paradigm shift in therapists' outlook towards teletherapy during the pandemic. Approximately 75% of therapists reported a surge in their interest in teletherapy, marking its status as a legitimate alternative to traditional therapeutic settings. This underscores the potential of telehealth in providing medical care and addressing the psychological challenges that arise during a pandemic [31].

While this study offers valuable insights, it is important to recognize its limitations. Foremost, the generalizability of the findings may be constrained due to the study's specific focus on individuals in Zamboanga City, Philippines. Also, the absence of a comparison group in the research design could impact the conclusiveness of the study's results. Consequently, the applicability of these findings to broader populations and settings should be interpreted with caution. Secondly, the study's reliance on self-reported measures, including assessments of depression, anxiety, stress, and COVID-19 preventive practices, introduces potential biases. Respondents' subjective perceptions can influence self-reporting and may not always align with clinical diagnoses. Thirdly, the study's scope primarily focused on the short-term impact of telehealth interventions during the early stages of the COVID-19 pandemic. Additionally, the study did not delve deeply into the experiences of healthcare providers who engaged in telehealth practices, an area that warrants further investigation. Exploring their perspectives, challenges, and training needs could offer critical insights into optimizing telehealth services and addressing potential limitations from the provider's viewpoint.

While acknowledging these limitations, we deliberately chose to extend telehealth services to a wide patient population, recognizing its critical importance during the peak of the COVID-19 pandemic. Furthermore, the study underscores the significance of adopting a whole-of-society approach in responding to these crises. These limitations, identified from the program's outset, should serve as valuable insights for future research and healthcare initiatives to optimize telehealth services for enhanced patient care and public health preparedness.

## Conclusion

This study was designed to determine the effects of a whole-of-society approach in telehealth education and consultation on patients confirmed to have COVID-19 but confined to government-sponsored isolation facilities. The study reviewed the effect of telehealth services on quarantine behavior, including maintaining social distancing, wearing appropriate face masks, sanitizing frequently handheld objects, self-monitoring for COVID-19 symptoms, and hand hygiene. Included also in this study was the assessment of patient’s levels of depression, stress, and anxiety from the time of their admission and discharge from the isolation facility. These patients were still followed up during their home quarantine in their community until after they were released to work and move about.

This study explores the potential of a whole-of-society approach in establishing a telehealth education and consultation program that might enhance isolated individuals' COVID-19 preventive practices and mental well-being. The findings show that it is possible to sustain and enhance quarantine practices while addressing participants' depression, anxiety, and stress. The study also suggests that telehealth education regarding mental health could potentially have a positive impact on the levels of depression, anxiety, and stress among isolated individuals. Furthermore, this potential improvement may be sustained even after discharge from the isolation facility and transitioning to home quarantine within the community.

With the implementation and findings of this study, telehealth education and consultation services can be applied to improve COVID-19 quarantine practices and the mental health of individuals in low-resource settings. It only needs a minimum of network logistics requirements, such as an internet connection. This study further concludes that telehealth services have the potential to address many critical challenges in providing continuous healthcare services to COVID-19-positive patients from admission into isolation facilities until discharge and reintegration into their community of residence.

## Data Availability

The datasets and materials are available from the corresponding author upon reasonable request.
